# Prevalence of depression, anxiety, adjustment disorders, and somatoform disorders in patients with age-related macular degeneration in Germany

**DOI:** 10.3205/000245

**Published:** 2017-02-09

**Authors:** Louis Jacob, Alexandra Spiess, Karel Kostev

**Affiliations:** 1 Faculty of Medicine, University of Paris 5, Paris, France; 2 IMS Health, Frankfurt, Germany

**Keywords:** age-related macular degeneration, depression, anxiety, adjustment disorder, somatoform disorder

## Abstract

**Aims:** The purpose of this study was to analyze the prevalence of depression, anxiety, adjustment disorders, and somatoform disorders in patients diagnosed with age-related macular degeneration (AMD) in Germany.

**Methods:** This study included 7,580 patients between the ages of 40 and 90 diagnosed with AMD between January 2011 and December 2014 in 1,072 primary care practices (index date). The last follow-up was in July 2016. We also included 7,580 controls without AMD, which were matched (1:1) to the AMD cases by age, sex, type of health insurance (private or statutory), physician, and Charlson comorbidity score as a generic marker of comorbidity. The outcome of the study was the prevalence of depression, anxiety, adjustment disorders, and somatoform disorders recorded in the database between the index date and the end of follow-up.

**Results:** The mean age among subjects was 75.7 years (SD=10.1 years), 34.0% were men, and 7.8% had private health insurance coverage. The Charlson comorbidity index was 2.0 (SD=1.8). Depression was the most frequent disease (33.7% in AMD patients versus 27.3% in controls), followed by somatoform disorders (19.6% and 16.7%), adjustment disorders (14.8% and 10.5%), and anxiety disorders (11.7% and 8.2%). Depression (OR=1.37, 95% CI: 1.27–1.47), anxiety (OR=1.50, 95% CI: 1.35–1.67), adjustment disorders (OR=1.50, 95% CI: 1.36–1.65), and somatoform disorders (OR=1.22, 95% CI: 1.12–1.32) were all positively associated with AMD.

**Conclusion:** Overall, a significant association was found between AMD and depression, anxiety, adjustment disorders, and somatoform disorders.

## Zusammenfassung

**Ziel:** In der vorliegenden Studie wurde die Prävalenz der psychiatrischen Komorbiditäten bei Patienten mit altersbedingten Makuladegeneration (AMD) und Personen ohne MD verglichen.

**Methoden:** Patienten mit einer gesicherten Diagnose der Makuladegeneration (ICD 10: H35.3) im Zeitraum 2011–2014 aus 1.072 allgemeinmedizinischen und internistischen Praxen wurden selektiert. Daten von 7.580 MD-Patienten und 7.580 Personen ohne MD aus allgemeinmedizinischen und internistischen Praxen in Deutschland wurden nach Matching für Alter, Geschlecht, Versicherungsstatus und Indexjahr (der letzte Eintrag in der Datenbank) retrospektiv analysiert. Der Anteil der Patienten mit einer gesicherten Diagnose von Depression, Angststörung, Reaktionen auf schwere Belastungen und Anpassungsstörungen oder somatoformen Störungen wurde ermittelt.

**Ergebnisse:** Patienten waren im Schnitt 75,7 Jahre alt (SD: 10,1), 34,0% waren männlich und 7,8% privat versichert. Charlson Comorbidity Index war im Schnitt 2,0 (SD: 1,8). Depression war die häufigste Diagnose (33,7% bei AMD-Patienten versus 27,3% in der Kontrollgruppe), gefolgt bei somatoformen Störungen (19,6% versus 16,7%), Reaktionen auf schwere Belastungen und Anpassungsstörungen (14,8% versus 10,5%), und Angststörungen (11,7% versus 8,2%).

Das Risiko für jede der untersuchten psychiatrischen Kodiagnosen war unter MD-Patienten nach der Adjustierung für Charlson Comorbidity Index insgesamt signifikant höher als bei Patienten ohne MD (Depression (OR: 1,37, 95% CI: 1,27–1,47), Angststörung (OR: 1,50, 95% CI: 1,35–1,67), Reaktionen auf schwere Belastungen und Anpassungsstörungen (OR: 1,50, 95% CI: 1,36–1,65), und somatoforme Störungen (OR: 1,22, 95% CI: 1,12–1,32)).

**Schlussfolgerung:** Makuladegeneration war mit stark erhöhtem Risiko für Depression, Angst-, Belastungs- sowie somatoformer Störung assoziiert.

## Introduction

Age-related macular degeneration (AMD), a disorder affecting older people, is one of the primary causes of irreversible loss of vision in industrialized countries [1], [2]. AMD is characterized by a deterioration of the macula, a region of the retina involved in central vision [3]. Advanced age, being female, smoking, and obesity are all risk factors for the development of this chronic condition [3]. In 2014, early AMD was discovered in 11.9% and late AMD in 0.2% of patients between the ages of 35 and 74 in Germany [2]. Such findings underline the high prevalence of AMD in this country, as well as the importance of personalized treatment and management for patients diagnosed with this ophthalmic disorder. 

AMD has a major impact on quality of life and thus is known to be positively associated with depression [4]. In 2007, a European study found that visual acuity had a significant impact on depression in people diagnosed with AMD, demonstrating that patients with low visual acuity were more frequently depressed than patients with high visual acuity [5]. In contrast, that same year, Sun et al. found no significant association between depressive symptoms and AMD in 2,194 American seniors [6]. Later, Jivraj and colleagues showed in Canada that depression was present in 21.3% of people diagnosed with AMD [7]. In line with this analysis, a 2014 UK systematic review that included 16 observational studies estimated that the rate of depression ranges between 15.7% and 44% and the rate of anxiety between 9.6% and 30.1% in patients with AMD [8]. Although such findings are of particular importance, little data is currently available on the relationship between AMD and these psychiatric disorders in Germany. 

Therefore, the goal of the present retrospective study was to analyze the prevalence of depression, anxiety, adjustment disorders, and somatoform disorders in patients diagnosed with AMD in Germany. 

## Methods

### Database

The Disease Analyzer database (IMS HEALTH) compiles drug prescriptions, diagnoses, and basic medical and demographic data from computer systems used in the practices of general practitioners and specialists [9]. The data are generated directly from computers in physicians’ practices via standardized interfaces and provide daily routine information on patients’ diseases and therapies. A practice transmits patient data stored in the physician’s computer to IMS on a monthly basis. Prior to transmission, the data are encrypted for data protection and contain, in similar scope and detail, the information in the files of patients in the doctor’s practice. The Disease Analyzer database provides a complete listing of all relevant patient details for each practice. The data obtained directly from the practices’ computers are checked for plausibility, linked to relevant additional information such as the price of a medicinal product, coded using ATC and ICD, saved, and updated on a monthly basis. The data bank includes only anonymized data in compliance with the regulations of the applicable data protection laws. The sampling method for the Disease Analyzer database is based on summary statistics from all doctors in Germany published yearly by the German Medical Association (Bundesärztekammer). The statistical unit of IMS uses these statistics to determine the panel design according to the following strata: specialist group, German federal state, community size category, and age of physician. The validity and representativeness of the Disease Analyzer database have been previously verified [9]. 

### Study population

**Figure 1:**
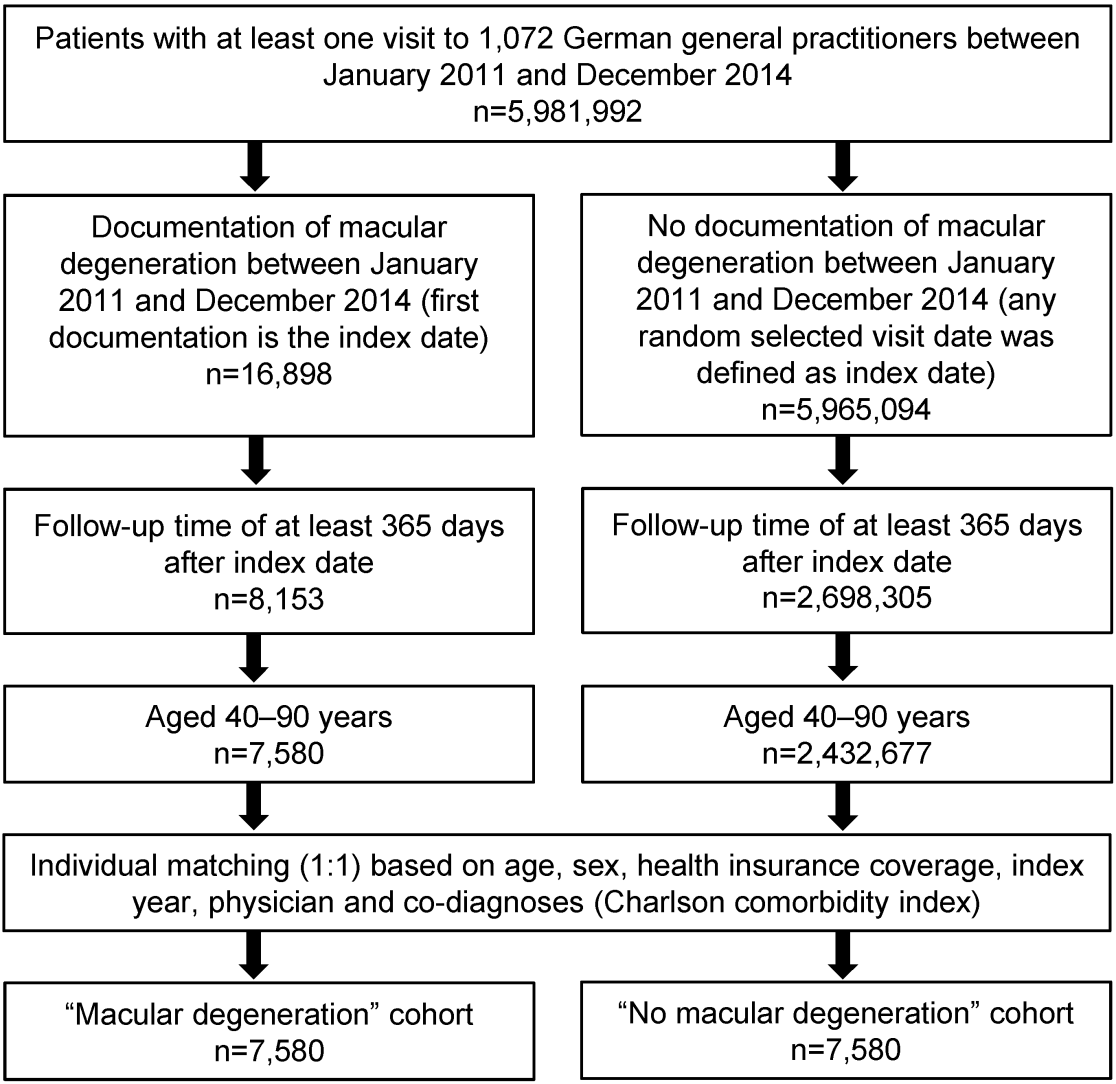
Selection of study patients

This study included 7,580 patients between the ages of 40 and 90 diagnosed with AMD (ICD 10: H35.3) between January 2011 and December 2014 in 1,072 primary care practices (index date). The last follow-up was in July 2016. AMD was initially diagnosed by ophthalmologists and subsequently documented by general practitioners. Finally, 7,580 controls without AMD (any randomly selected visit date was defined as the index date) were included and matched (1:1) to AMD cases by age, sex, type of health insurance (private or statutory), physician, and Charlson comorbidity score as a generic marker of comorbidity (Figure 1 (Fig. 1)) [10]. The Charlson comorbidity index (CCI) describes 22 comorbid conditions where each condition is assigned a score from 1 to 6 depending on the risk of death associated with each one. Clinical conditions are as follows: myocardial infarction, congestive heart failure, peripheral vascular disease, dementia, cerebrovascular disease, chronic lung disease, connective tissue disease, ulcer, chronic liver disease, diabetes, hemiplegia, moderate or severe kidney disease, diabetes with end organ damage, tumor, leukemia, lymphoma, moderate or severe liver disease, malignant tumor, metastasis, and AIDS [10]. CCI was included in order to estimate if a higher comorbidity status of patients can be associated with a higher risk of depression.

We only included the AMD cases and non-AMD controls in the analyses if they were followed for at least 365 days after the index date.

In Germany, no ethics votum is needed for studies based on anonymous epidemiological data. The IRB from IMS Health deemed ethical approval and written patient consent were not required as no ethics votum is needed for studies based on anonym epidemiological data.

### Study outcome

The outcome of the study was the prevalence of depression (ICD 10: F32, F33), anxiety (F41), reaction to severe stress, adjustment disorders (F43), and somatoform disorders (F45), recorded in the database between the index date and the end of follow-up. These diagnoses were based on primary care documentation. Moreover, the proportions of patients with one, two, and more than two different psychiatric diagnoses were estimated.

### Statistical analyses

Descriptive statistics were provided, and differences in characteristics of patients (AMD cases versus controls) were assessed using paired t-tests, Wilcoxon-tests for paired samples, or McNemar’s tests. The proportions of patients with depression, anxiety, adjustment disorders, and somatoform disorders were estimated in both the AMD and control groups. Logistic regression models (dependent variables: depression, anxiety, adjustment disorders, and somatoform disorders) were used to estimate the association between these disorders and AMD. Regression analysis was performed separately for each of the four disorders and for the risk of psychiatric multimorbidity, which is defined as at least two or at least three different psychiatric diseases. P-values <0.05 were considered statistically significant. The analyses were carried out using SAS version 9.3. 

## Results

**Figure 2:**
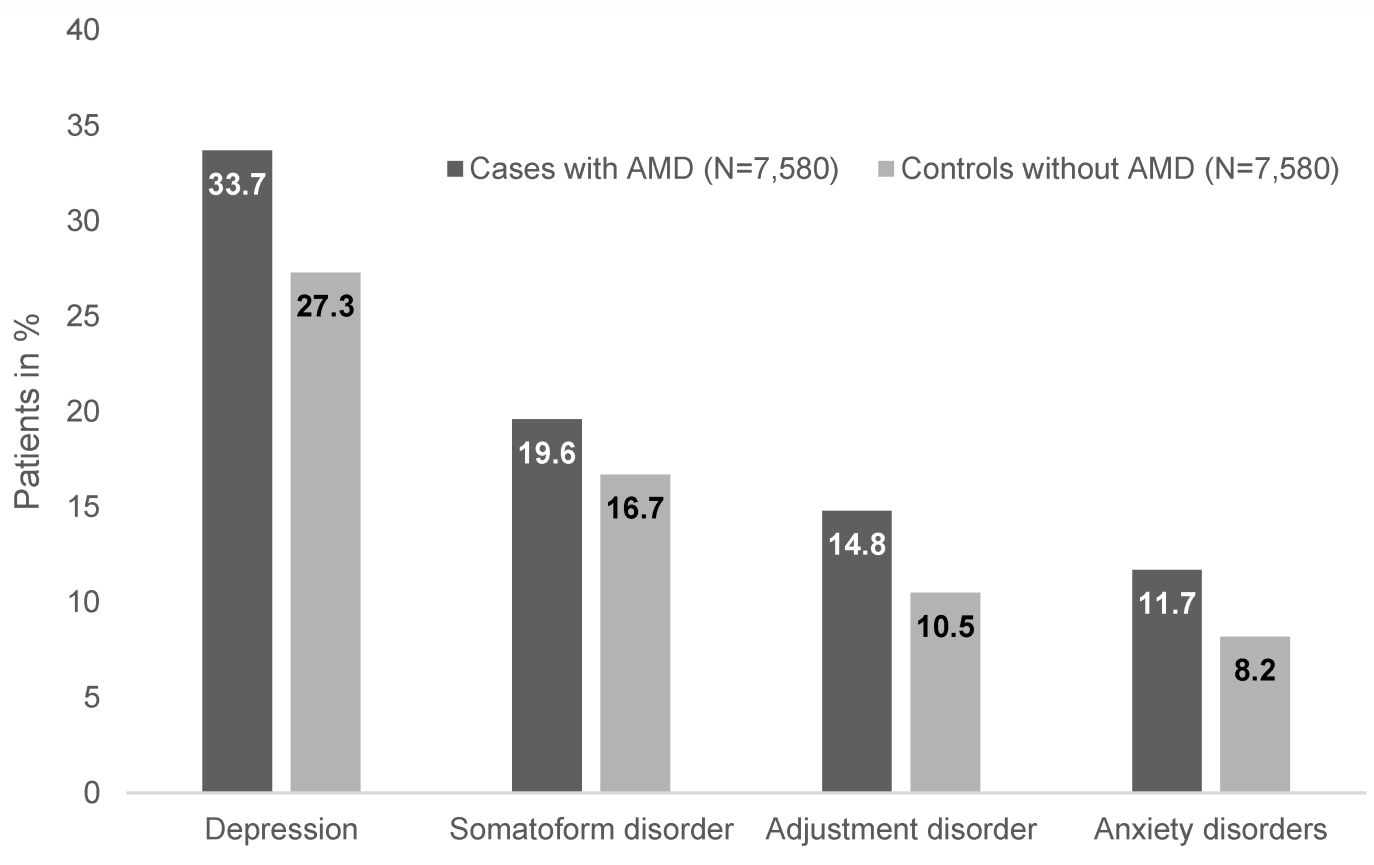
Prevalence of depression, anxiety, adjustment disorders, and somatoform disorders in patients with age-related macular degeneration (AMD) and controls in general practitioner practices

Patient characteristics are illustrated in Table 1 (Tab. 1). A total of 7,580 AMD cases and 7,580 non-AMD controls were included in the analysis. The mean age of the subjects was 75.7 years (SD=10.1 years), 34.0% were men, and 7.8% had private health insurance coverage. The Charlson comorbidity index was 2.0 (SD=1.8). Figure 2 (Fig. 2) displays the prevalence of the four psychiatric conditions in the AMD and control groups. Depression was the most common disease (33.7% in AMD patients and 27.3% in controls), followed by somatoform disorders (19.6% and 16.7%), adjustment disorders (14.8% and 10.5%), and anxiety disorders (11.7% and 8.2%). 

Furthermore, 27.2% of AMD patients and 24.2% of the controls were diagnosed with only one of the four psychiatric conditions; 14.3% of AMD patients and 11.6% of the controls were diagnosed with two conditions; and 21.8% of AMD patients and 16.4% of the controls were diagnosed with three or four psychiatric conditions.

The results of the multivariate logistic regression models are shown in Table 2 (Tab. 2). Depression (OR=1.37, 95% CI: 1.27–1.47), anxiety (OR=1.50, 95% CI: 1.35–1.67), adjustment disorders (OR=1.50, 95% CI: 1.36–1.65), and somatoform disorders (OR=1.22, 95% CI: 1.12–1.32) were positively associated with AMD. The risk of being diagnosed with at least two different psychiatric diagnoses (OR=1.43, 95% CI: 1.32–1.55) or at least three different psychiatric diagnoses (OR=1.63, 95% CI: 1.42–1.87) was significantly higher in AMD patients compared to the controls.

## Discussion

In the present retrospective study, which included 15,160 patients between 40 and 90, the prevalence of depression, anxiety, adjustment disorders, and somatoform disorders was higher in individuals with AMD than in controls. Multivariate logistic regression models further found that AMD was associated with an increased risk of developing these conditions (ORs ranging from 1.22 to 1.50).

Few authors have focused on the relationship between AMD and psychiatric disorders. In 2007, Augustin and colleagues estimated in a prospective study conducted in France, Germany, and Italy that the prevalence of depression increased with the severity of visual acuity impairment in patients diagnosed with AMD, with the rate of severe depression being 7.6% in the group with the lowest acuity [5]. In contrast, they also found that the prevalence of anxiety was unrelated to visual acuity and AMD progression. Using the Hospital Anxiety and Depression Scale, it was discovered that two items (“I still enjoy things I used to enjoy” and “I can enjoy a good book or radio or television program”) led to the identification of 95% of patients with moderate to severe depression. That same year, an American study that included 2,194 seniors resulted in conflicting findings, as depressive symptoms were not related to early or late AMD, whether the potential use of antidepressants was considered or not [6]. Although these last results are of particular interest, they need to be extrapolated and interpreted with caution. First, retinal photographs were taken nine years after baseline examination. Since depression was positively associated with mortality in the cohort used in this study [11], said mortality may have obscured the impact of AMD on depression. Second, almost 50% of eligible people did not participate in the study, potentially introducing a bias in the subsequent analysis. Third, patients may have been affected by AMD for years, and it is possible that they had adjusted to their ophthalmological condition and learned to cope with it prior to the depression assessment. 

In 2013, a Canadian study found that 21.3% of patients without a history of depression developed severe symptoms of this mental illness after AMD diagnosis [7]. In line with the work of Augustin and colleagues [5], low visual acuity increased the risk of developing depression. More recently, Dawson et al. conducted a systematic review of observational study data on the rate of anxiety and depression in people with AMD [8]. Assessment of 16 papers showed prevalence of depression to be between 15.7% and 44%, and prevalence of anxiety between 9.6% and 30.1% in AMD patients. The present German work corroborated these findings, as the proportion of depression was 33.7% and anxiety 11.7% in individuals affected by AMD, against 27.3% and 8.2% in controls, respectively. Therefore, the proportion of these two diseases is high in industrialized countries and underlines the importance of personalized management and treatment of people affected by AMD. For example, Rovner and colleagues discovered in a 2014 randomized clinical trial that the incidence of depressive disorders can be halved with integrated mental health treatment and low vision intervention in AMD individuals [12]. 

Another important result of this retrospective German study is that AMD was associated with adjustment disorders and somatoform disorders. This new finding may be explained by two hypotheses. As adjustment disorders and somatoform disorders are frequently found in patients affected by depression, it is possible that AMD does not have a direct impact on them but only on depression and anxiety. On the other hand, AMD is a chronic condition that may trigger the development of multiple psychiatric conditions. In 2012, Woo et al. found that patients with AMD are at a greater risk for cognitive impairment than non-AMD controls [13], suggesting that this ophthalmological disease is not only associated with depression and anxiety. Therefore, further studies are needed to analyze the potential effect of AMD on the overall mental health of the patient and particularly on adjustment disorders and somatoform disorders. 

The relationship between depression, anxiety, and AMD is complex and involves several factors. In 2002, Rovner and Casten hypothesized that valued activities are impaired in AMD patients with major visual function loss, indirectly increasing the risk of developing affective suffering and distress [14]. These findings were later corroborated in Australia and the Netherlands, as visual loss had a negative effect on activities of daily living, symptoms of depression, and feelings of anxiety [15], [16]. Nonetheless, one has to bear in mind that elderly patients with AMD are likely to be affected with other chronic conditions, which could also have an effect on such patients’ mental health. As a matter of fact, a 2010 meta-analysis estimated that current smoking status, higher body mass index, history of cardiovascular disease, hypertension, diabetes, history of cerebrovascular disease, and serum total and HDL cholesterol and triglyceride levels, were all risk factors for the diagnosis of AMD [17]. Interestingly, several of these risk factors were recently found to increase the odds of developing depression and anxiety in Germany [18], [19], [20]. As a result, it is possible that individuals with AMD are depressed not only because of the impairment of their visual acuity but also because of the impact of their other medical conditions on their daily lives. Again, this fact highlights the importance of interdisciplinary management of patients with AMD to prevent the development of depression and anxiety. 

This study has several limitations. First, the assessment of depression, anxiety, adjustment disorders, and somatoform disorders was based solely on ICD codes entered by general practitioners. This is a limitation of this study since different physicians may document diagnoses differently. Furthermore, patient care may also vary among practices. Second, visual acuity, which is known to have an impact on patients’ quality of life, was not recorded in the database. Third, data on socioeconomic status and quality of life were not available (i.e. marital status, alcohol/drug abuse, and stressful experiences), and such socioeconomic factors may also be predictors of psychiatric conditions. This limitation could have an impact on the results of regression analyses since adjusting for the missing variables is not possible. The possibility that patients with AMD have a different socioeconomic status compared to the control group cannot be excluded.

Overall, we found a significant association between AMD and depression, anxiety, adjustment disorders, and somatoform disorders. Further research is needed to gain a better understanding of the impact of AMD on these psychiatric conditions.

## Data

Data for this article are available from the Dryad Repository: http://dx.doi.org/10.5061/dryad.r7s04 [21].

## Notes

### Competing interests

Karel Kostev and Alexandra Spiess are employees of IMS Health. IMS Health (http://www.imshealth.de/sites/en/about-us/our-company) is a commercial research institute providing information, services and technology for the healthcare industry. Louis Jacob declares that he has no competing interests. 

## Figures and Tables

**Table 1:**
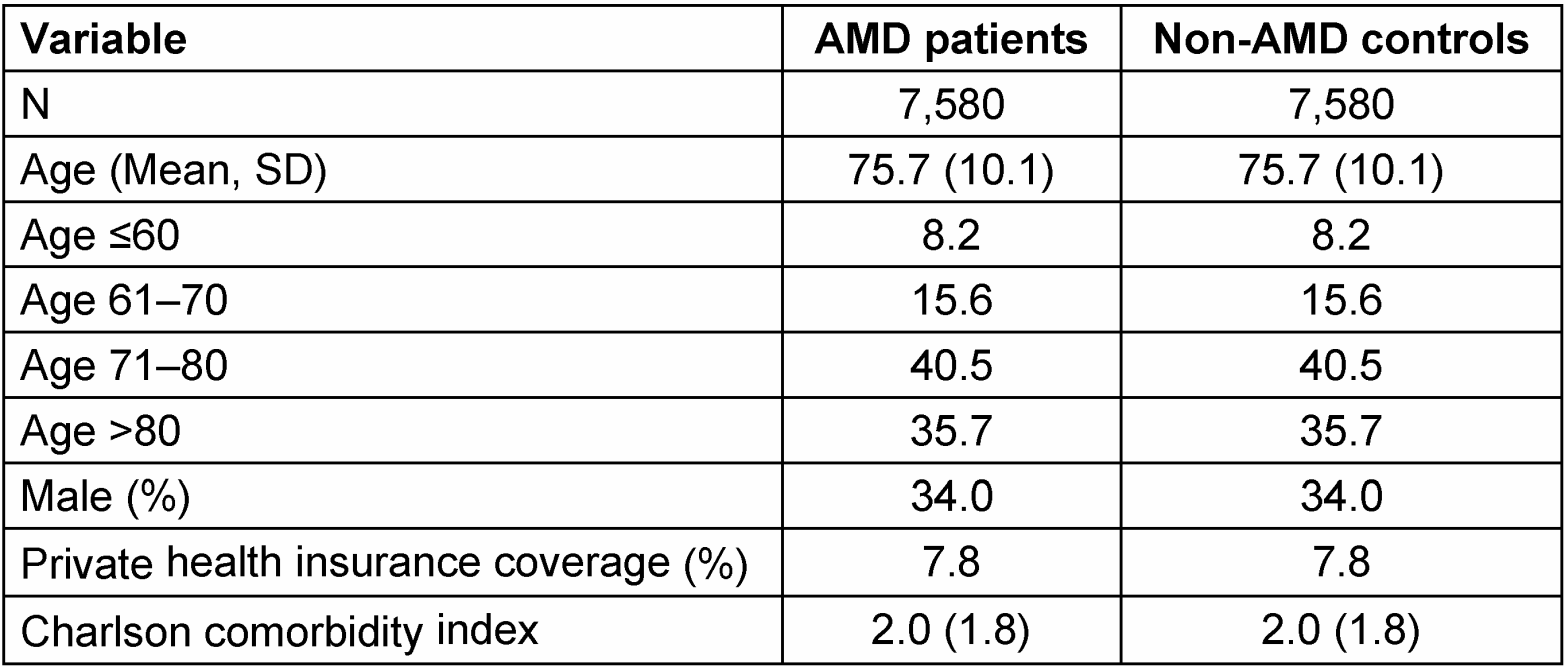
Characteristics of patients with age-related macular degeneration (AMD) and controls treated in German general practitioner practices after individual matching

**Table 2:**
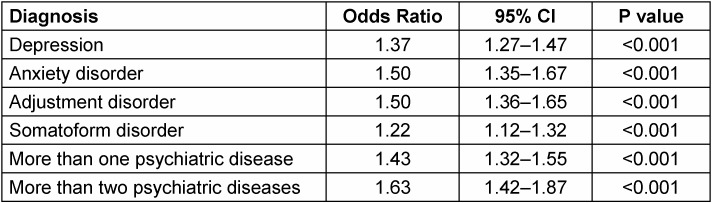
Association of age-related macular degeneration with depression, anxiety, adjustment disorders, and somatoform disorders (multivariate logistic regression)

## References

[1] Jager RD, Mieler WF, Miller JW (2008). Age-related macular degeneration. N Engl J Med.

[2] Korb CA, Kottler UB, Wolfram C, Hoehn R, Schulz A, Zwiener I, Wild PS, Pfeiffer N, Mirshahi A (2014). Prevalence of age-related macular degeneration in a large European cohort: results from the population-based Gutenberg Health Study. Graefes Arch Clin Exp Ophthalmol.

[3] Lim LS, Mitchell P, Seddon JM, Holz FG, Wong TY (2012). Age-related macular degeneration. Lancet.

[4] Casten RJ, Rovner BW (2013). Update on depression and age-related macular degeneration. Curr Opin Ophthalmol.

[5] Augustin A, Sahel JA, Bandello F, Dardennes R, Maurel F, Negrini C, Hieke K, Berdeaux G (2007). Anxiety and depression prevalence rates in age-related macular degeneration. Invest Ophthalmol Vis Sci.

[6] Sun C, Tikellis G, Klein R, Steffens DC, Larsen EK, Wong TY (2007). Depressive symptoms and age-related macular degeneration in older people: the cardiovascular health study. Ophthalmic Epidemiol.

[7] Jivraj J, Jivraj I, Tennant M, Rudnisky C (2013). Prevalence and impact of depressive symptoms in patients with age-related macular degeneration. Can J Ophthalmol.

[8] Dawson SR, Mallen CD, Gouldstone MB, Yarham R, Mansell G (2014). The prevalence of anxiety and depression in people with age-related macular degeneration: a systematic review of observational study data. BMC Ophthalmol.

[9] Becher H, Kostev K, Schröder-Bernhardi D (2009). Validity and representativeness of the “Disease Analyzer” patient database for use in pharmacoepidemiological and pharmacoeconomic studies. Int J Clin Pharmacol Ther.

[10] Quan H, Sundararajan V, Halfon P, Fong A, Burnand B, Luthi JC, Saunders LD, Beck CA, Feasby TE, Ghali WA (2005). Coding algorithms for defining comorbidities in ICD-9-CM and ICD-10 administrative data. Med Care.

[11] Ariyo AA, Haan M, Tangen CM, Rutledge JC, Cushman M, Dobs A, Furberg CD (2000). Depressive symptoms and risks of coronary heart disease and mortality in elderly Americans. Cardiovascular Health Study Collaborative Research Group. Circulation.

[12] Rovner BW, Casten RJ, Hegel MT, Massof RW, Leiby BE, Ho AC, Tasman WS (2014). Low vision depression prevention trial in age-related macular degeneration: a randomized clinical trial. Ophthalmology.

[13] Woo SJ, Park KH, Ahn J, Choe JY, Jeong H, Han JW, Kim TH, Kim KW (2012). Cognitive impairment in age-related macular degeneration and geographic atrophy. Ophthalmology.

[14] Rovner BW, Casten RJ (2002). Activity loss and depression in age-related macular degeneration. Am J Geriatr Psychiatry.

[15] Mathew RS, Delbaere K, Lord SR, Beaumont P, Vaegan, Madigan MC (2011). Depressive symptoms and quality of life in people with age- related macular degeneration. Ophthalmic Physiol Opt.

[16] Kempen GI, Ballemans J, Ranchor AV, van Rens GH, Zijlstra GA (2012). The impact of low vision on activities of daily living, symptoms of depression, feelings of anxiety and social support in community-living older adults seeking vision rehabilitation services. Qual Life Res.

[17] Chakravarthy U, Wong TY, Fletcher A, Piault E, Evans C, Zlateva G, Buggage R, Pleil A, Mitchell P (2010). Clinical risk factors for age-related macular degeneration: a systematic review and meta-analysis. BMC Ophthalmol.

[18] Drosselmeyer J, Jacob L, Rathmann W, Rapp MA, Kostev K (2017). Depression risk in patients with late-onset rheumatoid arthritis in Germany. Qual Life Res.

[19] Jacob L, Bleicher L, Kostev K, Kalder M (2016). Prevalence of depression, anxiety and their risk factors in German women with breast cancer in general and gynecological practices. J Cancer Res Clin Oncol.

[20] Konrad M, Bohlken J, Rapp MA, Kostev K (2016). Depression risk in patients with heart failure in primary care practices in Germany. Int Psychogeriatr.

[21] Kostev K (2017). Data from: Prevalence of depression, anxiety, adjustment disorders, and somatoform disorders in patients with age-related macular degeneration in Germany.

